# Complications After Craniofacial Surgery: A Review From 2012 to 2020

**DOI:** 10.7759/cureus.78625

**Published:** 2025-02-06

**Authors:** Maya Raghavan, Adrian A Ong, Michele M Carr

**Affiliations:** 1 Otolaryngology, University at Buffalo Jacobs School of Medicine and Biomedical Sciences, Buffalo, USA; 2 Plastic and Reconstructive Surgery, Norelle Health, New York, USA

**Keywords:** craniofacial anomaly, craniosynostosis, nsqip-p, pediatric otolaryngology, quality improvement, surgical complications, surgical outcomes

## Abstract

Objective

Craniofacial abnormalities require intensive surgical interventions associated with major risks. This study aimed to analyze how craniofacial surgical outcomes have changed over recent years.

Design

This was a retrospective, cross-sectional study.

Setting

Data was collected from the American College of Surgeons’ National Surgical Quality Improvement Program Pediatric (NSQIP-P) database.

Patients

A total of 1730 patients were identified between 2012 and 2020 by searching for CPT code 21175, "reconstruction, bifrontal, superior-lateral orbital rims, and lower forehead, advancement or alteration with or without grafts", in the primary procedure column. Variables of interest included total length of hospital stay (LOS), readmission, and reoperation.

Main outcome measures

The primary outcome measured was the incidence of postoperative complications.

Results

The study included 1730 patients, with a mean age of 1.8 + 2.7 years. The most common comorbidities were structural CNS abnormalities (24%), developmental delay or impaired cognitive status (17%), and prematurity (16%). Nearly all patients (N = 1721, 99.5%) had a comorbidity, 1270 (73%) patients required blood transfusion, and 68 (4%) patients experienced another type of complication. The complication rate decreased over time, with 80% of patients experiencing a complication in 2012 and 72% in 2020 (p = 0.008). The mean LOS steadily decreased over that time from 5.3 days to 3.6 days (p < 0.001). The time from operation to discharge also decreased at a similar rate to the total LOS (p < 0.001). There was no significant change in the incidence of readmission (1.9%) or reoperation (2.4%) over time.

Conclusions

The LOS and incidence of complications have decreased over time, suggesting improvements in craniofacial surgery practices.

## Introduction

Craniofacial anomalies occur in the general population at an incidence of 0.2-0.5 per 1000 births and account for one-third of congenital abnormalities [1]. The majority of craniofacial anomalies, specifically craniosynostosis, are considered isolated defects with a wide range of etiologies [2]. There are over 200 syndromes that can result in craniosynostosis, although only 20% of anomalies are due to known genetic conditions [3]. The diversity of causes and types of craniofacial anomalies has necessitated an array of procedures for management, making treatment and outcome assessment particularly case-specific and complex [4]. Techniques in craniofacial surgery have evolved over time alongside more specialized and demographic-specific risk management.

Craniofacial anomalies typically require early and intensive surgical intervention associated with major risks [5,6]. The incidence of surgical complications has been seen to increase with age, which makes operating before 12 months of age a priority in most cases [7]. Blood loss requiring transfusion is the most common complication of pediatric craniofacial surgery, which can result in serious transfusion-related side effects, sometimes leading to mortality [8]. The success of craniofacial surgery has increasingly relied on a coordinated multidisciplinary team to address the complexity of the surgery itself, along with the complications and long-term follow-up care [9]. However, high complication rates continue to pose a challenge in craniofacial surgery, requiring special care and consideration in the pediatric population [6]. The goal of this study was to assess how craniofacial surgical outcomes have changed over recent years.

This article was previously presented as a meeting abstract at the AAO-HNS Annual Meeting on September 13, 2022.

## Materials and methods

Study design

A retrospective study was conducted using the American College of Surgeons National Surgical Quality Improvement Program Pediatric (ACS NSQIP-P) database from 2012 to 2020 [10]. The study was exempt from approval by the University at Buffalo Institutional Review Board due to the use of publicly available deidentified data. The authors did not have access to information that could identify participants during or after data collection. Informed consent was not required. Data was accessed on January 24, 2022.

Data was available for over 150 surgical pre-, intra-, and postoperative variables. ACS NSQIP-P uses a specialized system of in-person clinical reviewers and specially designed software to collect data. Data collection and review practices are standardized to achieve high quality and consistency. Database data collection methods are detailed in the ACS NSQIP-P User Guide.

The Participant Use Data File contains 128,395 cases from 148 sites in 2020, 132,881 cases from 141 sites in 2019, 119,486 cases from 127 sites in 2018, 113,922 cases from 109 sites in 2017, 101,887 cases from 101 sites in 2016, 84,056 cases from 80 sites in 2015, 68,838 cases from 50 sites in 2014, 63,387 from 56 sites in 2013, and 51,008 cases from 50 sites in 2012. 

For this study, cases were identified using Current Procedural Terminology (CPT) code 21175 for “reconstruction, bifrontal, superior-lateral orbital rims, and lower forehead, advancement or alteration with or without grafts” under “repair, revision, and/or reconstruction procedures on the head”. Patients were eligible for inclusion if CPT code 21175 was the primary procedure from 2012 to 2020. All patients under 18 years of age were eligible for inclusion. Variables of interest included patient demographics, perioperative comorbidities and risk factors, and specific postoperative complications, including surgical site infection, bleeding, and unplanned intubation. Total length of hospital stay (LOS), time from operation to discharge, and readmission rates were also evaluated. 

Statistical analysis

Data was described and analyzed using Stata 15 (StataCorp LLC, College Station, TX, USA). Nonparametric data was analyzed using Kruskal-Wallis tests. Categorical variables were compared using chi-squared or Fisher’s exact tests depending on the sample size. Linear and logistic regression models were built with stepwise modeling. Significance was determined as p < 0.05 because this study was defined as an exploratory analysis [11].

## Results

This study included 1730 patients, with a mean age of 1.8 + 2.7 years (range 0-17.5). Gender distribution showed no significant change over the years, while race distribution changed significantly, though without a clear trend (p < 0.001). The increasing amount of missing data for race, from 9% in 2012 to 23% in 2020, hindered the analysis of potential changes in race distribution over time. Demographic information is provided in Table 1.

**Table 1:** Demographic information from 2012 to 2020 (N=1730) ^a^Other includes American Indian/Alaska Native and Native Hawaiian/Other Pacific Islander race.

Demographics	Value
Age	
Mean ± SD	1.8 ± 2.7
Median (95% CI)	0.90 (0.87-0.92)
Gender, N (%)	
Male	1027 (59)
Female	703 (41)
Race, N (%)	
White	1203 (70)
Black or African American	146 (8)
Asian	48 (3)
Other^a^	23 (1)
Unknown	306 (18)

Nearly half (N = 790, 46%) of the patients had a comorbidity not including congenital malformation. The most common comorbidities were structural CNS abnormalities (24%), developmental delay or impaired cognitive status (17%), and prematurity (16%). The likelihood of comorbidity increased with older age (OR, 1.1; p < 0.001). The percentage of patients with comorbidities fluctuated significantly from 2012 to 2020 without a directional trend (p = 0.001; Table 2). The likelihood of postoperative complications increased if a patient had a comorbidity (OR, 1.3; p = 0.012). Blood transfusions were required by 1270 (73%) patients, and 68 (4%) patients experienced another type of complication. A total of 89 complications other than bleeding were documented (Table 2). The incidence of postoperative complications decreased over time from 80% of patients in 2012 to 72% in 2020 (Table 3). Regression showed a significantly decreased complication rate over time (p = 0.008). There was no significant change in the incidence of any specific complication over time.

**Table 2:** Postoperative complications (N=1730) CVA: cerebral vascular accident, VT: ventricular tachycardia.

Complications	N (%)
Superficial incisional surgical site infection	21 (1.2)
Deep incisional surgical site infection	7 (0.4)
Organ/space surgical site infection	15 (0.9)
Deep wound disruption/dehiscence	8 (0.5)
Pneumonia	1 (0.1)
Unplanned intubation	7 (0.4)
Urinary tract infection	2 (0.1)
Stroke/CVA	4 (0.2)
Seizure	9 (0.5)
Bleeding/transfusions	1270 (73.4)
Cardiac arrest	2 (0.1)
VT requiring therapy	1 (0.1)
*Clostridioides difficile* colitis	4 (0.2)
Systemic sepsis	6 (0.3)
Septic shock	1 (0.1)
Central line-associated bloodstream infection	1 (0.1)

**Table 3:** Preoperative comorbidities and postoperative complications by year ^a^Chi-squared test. ^†^Not including congenital malformation.

Variable	Year									p-value
	2012	2013	2014	2015	2016	2017	2018	2019	2020	
Any comorbidity^†^, N (%)	77 (41)	116 (60)	3 (38)	89 (45)	110 (48)	89 (39)	97 (40)	90 (45)	110 (46)	0.001^a^
Any complication, N (%)	190 (80)	192 (79)	8 (88)	148 (74)	174 (75)	163 (72)	172 (72)	147 (73)	173 (72)	0.33^a^

The mean LOS significantly decreased from 5.3 days in 2012 to 3.6 days in 2020 (p < 0.001). Perioperative data is described in Table 4. Both preoperative comorbidities and postoperative complications were correlated with increased LOS (both p < 0.001). The mean time from operation to discharge decreased from 5.6 days in 2012 to 3.5 days in 2020 (p < 0.001). The mean anesthesia time and operative time both differed significantly from year to year, although they did not show a directional trend (p < 0.001 and p = 0.003, respectively). There was no significant change in the incidence of readmission (1.9%) or reoperation (2.4%) over time.

**Table 4:** Perioperative outcomes by year ^a^Kruskal-Wallis test. ^b^Fisher’s exact test.

Perioperative 0utcomes	Year									p-value
	2012	2013	2014	2015	2016	2017	2018	2019	2020	
Duration of anesthesia ± SD (min)	367 ± 127.7	351.3 ± 115.4	402 ± 65.6	362.7 ± 103.5	325.1 ± 99.9	343.93 ± 101.7	340 ± 105.9	364.9 ± 92.2	356.3 ± 103.7	<0.001^a^
Operative time ± SD (min)	249.8 ± 112.6	245.1 ± 99.8	282.5 ± 64	251.4 ± 91.2	219.8 ± 91.2	236.8 ± 90.5	230.6 ± 94.1	247.4 ± 82.7	244.5 ± 88.9	0.003^a^
Time from operation to discharge ± SD (days)	5.6 ± 8.3	4.2 ± 1.2	3.9 ± 1.1	4.5 ± 2.4	4.1 ± 1.7	4.0 ± 2.4	4.0 ± 2.8	4.1 ± 2.6	3.5 ± 1.2	<0.001^a^
Length of hospital stay ± SD (days)	5.3 ± 4.4	4.2 ± 1.4	3.9 ± 1.1	4.5 ± 2.7	4.3 ± 2.1	4.1 ± 3.1	4.0 ± 2.8	4.1 ± 2.6	3.6 ± 1.5	0.001^a^
Related readmission (%)	3	2	0	7	4	5	5	3	4	0.86^b^
Related reoperation (%)	5	3	0	5	6	8	5	6	4	.93^b^
Still in hospital at 30 days (%)	1	0	1	0	0	1	0	0	1	.013^b^

Of the patients, 1063 (62%) underwent concurrent procedures, with 911 of those undergoing concurrent craniotomy or craniectomy. Additionally, 251 (15%) patients underwent more than one concurrent procedure, and 1228 (71%) patients underwent 96 unique other procedures apart from the primary craniofacial surgery. 

## Discussion

Our data suggest that outcomes in craniofacial surgery have improved in recent years. This progress may be attributed to improved safety practices. Multidisciplinary teams are increasingly being relied on to coordinate care throughout patients’ stays in the hospital, even long-term follow-up and continued care [12]. With the establishment of a “craniofacial team,” healthcare professionals are more specifically trained to control and plan for the unique challenges and risks inherent to craniofacial surgery [5,9].

The 2017 American Association of Oral and Maxillofacial Surgeons (AAOMS) Clinical Practice Guidelines for cleft and craniofacial surgery compiled a list of 27 general surgical risks and complications, including unplanned admission to the intensive care unit, facial/trigeminal nerve dysfunction, infection, postoperative hemorrhage, repeat surgery, and residual deformity. Each craniofacial abnormality also has its own specific set of surgical risks [6]. These guidelines added that special consideration must be given to pediatric cleft and craniofacial surgery because continuing facial growth in children complicates long-term outcomes. Assessing the optimal timing and ideal procedures for pediatric craniofacial surgery must consider facial growth, middle ear function, speech and airway development, and psychosocial distress. Surgical techniques are wide-ranging to account for the diversity in craniofacial abnormalities and the patient-specific primary goals of surgery, which makes standardizing procedure and risk assessment difficult [4].

The Enhanced Recovery After Surgery (ERAS) protocol has been used in many surgical fields, with more recent utilization in craniofacial surgery [13]. It entails preoperative, intraoperative, and postoperative interventions to prevent surgical complications. Interventions include preoperative erythropoietin (EPO) supplementation, intraoperative use of tranexamic acid (TXA) and cell saver, and postoperative scheduled acetaminophen and ibuprofen regimens to reduce opioid use [14]. Srivatsa et al. reported multicenter data detailing patient management after open craniosynostosis repair [15]. Without the ERAS protocol, the mean postoperative stay was 3.5 days, and 95% of patients used opioids. Improved surgical outcomes have been seen in single-institution studies in the past 10 years with the implementation of the ERAS protocol [13-16].

Knackstedt and Patel detailed changes in surgical outcomes after their institutions began using the ERAS protocol in 2013 for pediatric fronto-orbital advancement in craniosynostosis [14]. They implemented preoperative EPO supplementation in patients under 18 months of age, preparation of packed red blood cells divided into smaller aliquots prior to surgery, increased operating room temperature and warming mattresses, cell-saver technology, TXA, dexmedetomidine drip, scheduled acetaminophen and ibuprofen, and quickly titrated fluids according to postoperative urine output. They saw a significantly decreased LOS, decreased narcotic utilization, and decreased blood loss. With the ERAS protocol, the mean LOS decreased from 3.6 to 2.3 nights.

Martin et al. found that the use of TXA alone, independent of other ERAS protocol interventions, resulted in significantly decreased blood loss, blood volume transfused, and LOS in non-syndromic single-suture synostosis repair [16]. More unified efforts to establish the use of the ERAS protocol in craniofacial surgery began in 2019 among members of the American Society of Craniofacial Surgeons and the American Society of Maxillofacial Surgeons [13]. Although Wu et al. found no decrease in LOS associated with the ERAS protocol between 2007 and 2014 in a database study, single-center studies show promise for improving outcomes if the ERAS protocol is more widely implemented [17].

Additionally, the use of minimally invasive endoscopic surgery in pediatric populations could result in dramatically improved surgical outcomes. Riordan et al. showed that early operation using a minimally invasive approach in patients normally requiring fronto-orbital advancement led to transfusion rates as low as 6% with 4% complication incidence [18]. This is especially noteworthy when compared to our study’s results, showing a transfusion rate of over 70% and complication incidences between 72% and 88%. Minimally invasive endoscopic surgery is currently recommended for patients under three months of age, which limits its utility since patients quickly age out and still require open surgery [18].

Bruce et al. showed that complications increased with increasing age at operation, further supporting the importance of early repair [7]. The fewest complications were seen in 0-to-6-month-old patients. The mean age at operation in this study was 1.8 years, with over half of patients under the age of one year. This finding demonstrates that early operation is currently common practice, although there is a wide range of ages at operation. Further study would need to elucidate the risks and benefits of prioritizing the use of endoscopic surgery when determining the optimal timing for intervention.

Limitations

The main limitations of a retrospective database study include generic short-term analysis and human error in data collection and coding. Specific in-depth research is limited by the generic nature of the variables. When using a database, there is also the inherent inability to control for confounding variables. The hospitals voluntarily participating in NSQIP do not form a nationally representative sample, and the hospitals included tend toward higher-acuity tertiary care settings biased toward more complicated cases. Additionally, the NSQIP database collects data for only 30 days after surgery, so analysis is limited to early postsurgical outcomes. By defining our study parameters using one CPT code for a specific type of craniofacial surgery, the extrapolation of results to other surgery outcomes in this diverse field is limited. Retrospective database study is best utilized as a primary exploration of trends that can provide direction for future in-depth analysis.

## Conclusions

The LOS, time from operation to discharge, and incidence of postsurgical complications all significantly decreased between 2012 and 2020. Changing practices in this field, including advancements in surgical techniques, patient management strategies, and perioperative care, may contribute to improved outcomes over time. Further research is necessary to better understand recent changes in practice and their direct impact on craniofacial surgical outcomes.
